# Ancient Eastern Methods

**Published:** 1907-04-27

**Authors:** 


### The Evolution of Medicine.

### ANCIENT EASTERN METHODS.

### I.—THE BARBER-SURGEON OF THE EAST.

The "Bagh O Bahm" is an Urdu classic, containing a number of stories something after the fashion of the "Arabian Nights." The book was first published in its present form about the year 1800, but the tales are of much earlier origin. The scene of the majority of them is laid in the Persian Gulf and in Turkey, that of a fair proportion in India. Some of the stories deal with the treatment of wounds and diseases, and thus incidentally throw light upon the manners and methods of the practitioners of medicine and surgery, whose practice appears to have been based upon a curious combination of science and superstition.

#### The Hajam and his Methods.

In one of the stories we are introduced to the barber (hajam), who frequently, as was formerly the case in England, combined the practice of surgery with the pursuit of his proper vocation. The hajam must be distinguished from the surgeon proper (jarrah, dresser of wounds) and from the professional physician (hakeem), both of whom were no doubt more highly esteemed as specialists in their profession. In the story in question, one of the characters, the first dervish, relates that he found a lovely lady, a princess, lying wounded in a box beneath the wall of a certain city. He went to look for a jarrah, but was recommended to the house of a hajam. "There is a hajam," he was told, "ripely skilled in the healing of wounds and in the science of hakeem. In this work he is so ripely skilled that if a corpse be taken to him, by the orders of God he will make a plan by which it will at once rise up alive."

The name of the Barber-surgeon was Isa, and he did not belie his reputation. The Dervish hastened to the house and there found the hajam, a white-bearded man, sitting in a portico superintending the labours of several other men who were pounding materials for the preparation of an ointment. Apparently Isa was in a position to make his attendance on a case a matter of favour, for the dervish expressly says "I flattered him into going by treating him with great respect." Incidentally he told a good many falsehoods about the case, from which it will be observed that in one respect men were pretty much as they still are. The entreaties of the dervish prevailed. "The surgeon, Isa," he tells us, "was an exceedingly kind-hearted and God-worshipping man. He had compassion on my humble words and came to this house with me. The instant he looked on the wounds he gave me consolation. He said by the mercy of God this lady's wounds will be made to heal in forty days"—forty days was usually occupied by any doctor in the cure of any kind of case. "I will make her take the bath of cure."

#### His Method of Treatment.

The treatment is thus described :

"This man of God washed all the wounds in *neem* leaf water and made them clean. Those that were fit to receive the thread he sewed up. As for the remaining gashes, he took a small bag out of his wallet and placed bandages over some, and having put plaister over others he bound them with bandages, and said with extreme kindness : 'I will continue to come twice (daily). Be very careful that she make no movement by which the stitches may be torn. Give chicken soup for her food; pour it down her throat, and frequently give extract of musk willow with rose-water that strength may remain.' Having said this, he requested permission to depart."

#### His Social Status.

The position of the barber-surgeon of the East seems to have been superior to that of his contemporaries in the West. He sits at his ease in the portico of his house watching his men at work, and must be approached with respect and entreated to attend. He is described as kind-hearted and religious. He appears also to have had not a little skill in his subsidiary profession. There is no suggestion of charms or magic such as were probably employed by other Eastern practitioners of the time. He examines his patient, gives a prognosis, comforts the friends, and discriminates in his treatment of various kinds of wounds, the remedies for which he carries with him in his wallet. He understands the use of sutures, and knows that all wounds are not suitable for suture. He impresses on the friend of the patient the necessity of rest for healing and of food and stimulants to keep up the patient's strength. His present-day successors could do little more than Isa did for this poor lady. His contemporaries would probably have ordered boiling oil and bleeding. His manners are courteous and polite; he asks permission to depart—an Eastern courtesy—and he receives gratitude and ceremonious respect from the patient's friends. The dervish displays his gratitude and respect by words and actions. "I stood," he says, "with joined hands and said, 'By your kindness my life is saved; but for you nothing was left me but to die. May God's peace rest on you.' I gave him attar and pam, and permission to depart." Still in the East attar is sprinkled on, and pam given to, the departing friend or guest whom it is desired to honour.

The dervish nursed the lady with devotion. The hajam paid frequent visits. "In a short time the wounds filled up and formed grapes"—an excellent description of healthy granulations. "She took the bath of cure." And the hajam received a liberal fee. "I piled up robes of honour and golden coins before Isa the barber," says the chronicler in concluding his narrative. What was the exact amount of the hajam's fee we are left to imagine; definite statistical information was, apparently, as distasteful to the dervish as it is to some modern chroniclers.

#### Some of his Remedies.

The *neem* referred to in this narrative is a large tree, common in India and resembling the English ash. Both a decoction and a poultice of its leaves, which are bitter to taste, are held in great estimation by the natives as a remedy for wounds and sores. The use of rose water requires no explanation. It was very frequently ordered, and was a favourite in the Indian Pharmacopœia. As to the musk willow, the present writer is unable to speak of his personal knowledge; it is said to be a scented variety of willow. The "bath of cure" or convalescence appears to have been ceremonial rather than curative, inasmuch as it is always spoken of as being taken after the patient has recovered from his wounds. In a case such as that which has been recorded, it adds the indispensable touch of Oriental ceremony to a course of treatment in other respects thoroughly practical.

